# Supplementary material for: The Ameliorative Effect of Litsea martabanica (Kurz) Hook. f. Leaf Water Extract on Chlorpyrifos-Induced Toxicity in Rats and Its Antioxidant Potentials
Source: Foods. 2024 May 28;13(11):1695. doi: 10.3390/foods13111695 (PMC11172329; doi:10.3390/foods13111695)
Supplement: Supplementary file 1 [file foods-13-01695-s001.zip › foods-3007449-supplementary.pdf]

foods

Supplementary Data

The Ameliorative Effect of *Litsea martabanica* (Kurz) Hook. f. Leaf Water Extract on Chlorpyrifos-Induced Toxicity in Rats and Its Antioxidant Potentials

Supplementary Figure S1: TLC Chromatogram of *Litsea martabanica* water extract

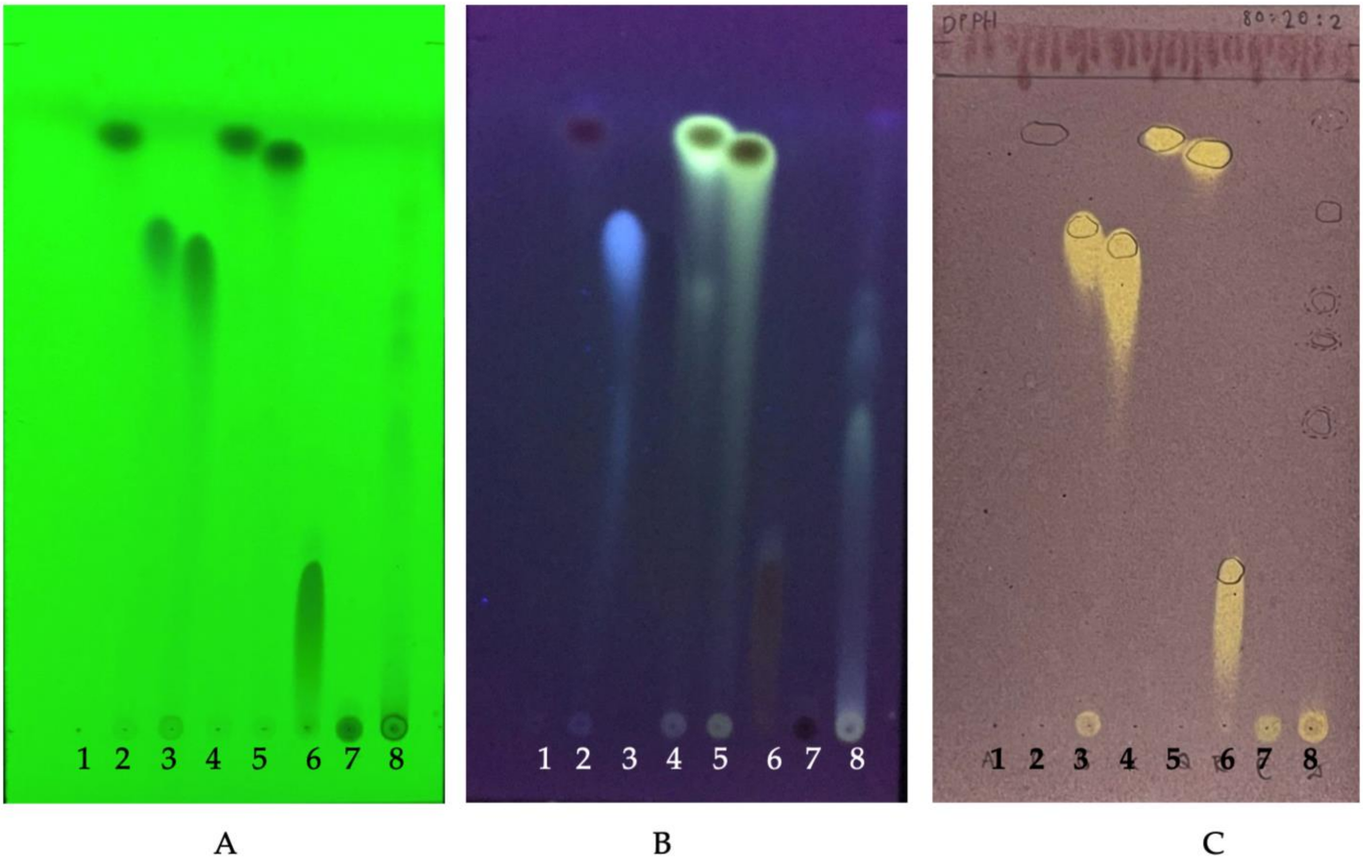

Figure S1. TLC Chromatogram of the water extract of *L. martabanica.* (A) UV 254 nm, (B) UV 366 nm, and (C) DPPH spraying reagent. (1 = Apigenin; 2 = Caffeic acid; 3 = Gallic acid; 4 = Kaempferol; 5 = Quercetin; 6 = Rutin; 7 = Ellagic acid; 8= *Litsea martabanica* water extract)
